# Integrated analysis of miRNAs and mRNA profiling reveals the potential roles of miRNAs in sheep hair follicle development

**DOI:** 10.1186/s12864-022-08954-2

**Published:** 2022-10-22

**Authors:** Junmin He, Xixia Huang, Bingru Zhao, Guifen Liu, Yuezhen Tian, Guoping Zhang, Chen Wei, Jingyi Mao, Kechuan Tian

**Affiliations:** 1 Key Laboratory of Livestock and Poultry Multi-Omics of MARA, Institute of Animal Science and Veterinary Medicine, Shandong Academy of Agricultural Sciences, Jinan, China; 2 College of Animal Science, Xinjiang Agricultural University, Urumqi, China; 3 College of Animal Science and Technology, Nanjing Agricultural University, Nanjing, China; 4 Institute of Animal Science, Xinjiang Academy of Animal Sciences, Urumqi, China

**Keywords:** Hair follicle, Merino sheep, miRNA–mRNA, Dermal fibroblasts, Western blot

## Abstract

**Background:**

Merino sheep exhibit high wool production and excellent wool quality. The fleece of Merino sheep is predominantly composed of wool fibers grown from hair follicles (HFs). The HF is a complex biological system involved in a dynamic process governed by gene regulation, and gene expression is regulated by microRNAs (miRNAs). miRNA inhibits posttranscriptional gene expression by specifically binding to target messenger RNA (mRNA) and plays an important role in regulating gene expression, the cell cycle and biological development sequences. The purpose of this study was to examine mRNA and miRNA binding to identify key miRNAs and target genes related to HF development. This will provide new and important insights into fundamental mechanisms that regulate cellular activity and cell fate decisions within and outside of the skin.

**Results:**

We analyzed miRNA data in skin tissues collected from 18 Merino sheep on four embryonic days (E65, E85, E105 and E135) and two postnatal days (D7 and D30) and identified 87 differentially expressed miRNAs (DE-miRNAs). These six stages were further divided into two longer developmental stages based on heatmap cluster analysis, and the results showed that DE-mRNAs in Stage A were closely related to HF morphogenesis. A coanalysis of Stage A DE-mRNAs and DE-miRNAs revealed that 9 DE-miRNAs and 17 DE-mRNAs presented targeting relationships in Stage A. We found that miR-23b and miR-133 could target and regulate ACVR1B and WNT10A. In dermal fibroblasts, the overexpression of miR-133 significantly reduced the mRNA and protein expression levels of ACVR1B. The overexpression of miR-23b significantly reduced the mRNA and protein expression levels of WNT10A.

**Conclusion:**

This study provides a new reference for understanding the molecular basis of HF development and lays a foundation for further improving sheep HF breeding. miRNAs and target genes related to hair follicular development were found, which provided a theoretical basis for molecular breeding for the culture of fine-wool sheep.

**Supplementary Information:**

The online version contains supplementary material available at 10.1186/s12864-022-08954-2.

## Background

Subo Merino (SBM) sheep are breed reared in China that produce superfine wool. Wool fiber diameter is typically 17–19 μm, which is larger than the wool quality count of 80 [1] and greatly impacts the fine-wool sheep industry. SBM sheep are an essential element of animal husbandry in China. Hair follicles (HFs), which control sheep wool growth and development, are thin structures with a complex morphology and a periodic growth pattern [2]. In addition to the epithelial and hypodermal layers, hairs also include connective tissue sheaths, inner root sheaths, outer root sheaths, hair bulbs, and hair shafts [3]. The HFs of fine-wool sheep are composed of primary follicles (PFs) and secondary follicles (SFs); PFs appear early, whereas SFs appear late [4]. Compared with PFs, SFs have a more obvious effect on wool yield and quality. The larger the proportion of SFs, the smaller the wool fiber diameter is [5]. The most important factor affecting HF density is SF redifferentiation, which can be increased to improve wool fineness. It has been shown that HFs present distinct characteristics of gene expression and functions [6]. Merino wool follicles have been described in detail, and it is well established that no new follicles appear after birth [7–12]. Within 75 days of gestation, the first follicles begin to form in sheep fetus PFs, and they produce fibers by 90 days [7, 8, 13]. It is not until approximately 85 days of gestation that SFs begin to develop. At approximately 105 days of gestation, secondary-derived follicles (SDs) begin to branch off from SFs [14]. Because HFs fully mature before birth, their numbers do not increase after birth. A study by Zhao et al. [15] from our group investigated the morphogenesis of ovine HFs at six developmental stages based on histomorphological observations. They found that placodes and dermal condensate started to form at E65, indicating the induction of HFs. At E85, the number of PFs increased, and SFs began to form. SFs began to differentiate at E105, and the number of SDs began to increase at E135. HFs had matured and showed a complete structure at E135, and most of the hair shafts had emerged through the epidermis [15].

Hair bud formation in the epidermis is initiated by signals from the dermis, followed by the release of TGF-β [16], EDA/EDAR [17], NF-κB [18, 19], Noggin/Lef-1 [20], shh [21], BMP-2/4/7 [22], WNT/β-catenin [23–27], and FGF in hair buds, which induces dermal fibrogenic cells to form the dermal papillae that regulate keratinocyte activity [28]. Embryonic fibroblasts are essential for HF morphogenesis and wound closure, but they are subsequently unable to induce new HFs unless embryonic programs are reactivated. Fibroblasts are the main resident cells of the skin dermis and participate in embryonic HF morphogenesis and wound healing, since extracellular matrix is remodeled and collagen is deposited in the wound bed as a result of their activity [29]. Importantly, dermal fibroblasts originate from two distinct developmental lineages with unique functions that differentially mediate the response to epidermal signals such as Hedgehog signaling [30]. The cells of dermal papillae release a second signal to stimulate epithelial cell proliferation, which initiates differentiation and results in a complete HF structure. Considering the complexity of HFs, studies on fetal skin have been valuable for identifying all candidate genes that appear to be developmentally regulated.

The majority of genetic variants discovered in genome-wide association studies (GWAS) occur in noncoding RNAs [31]. Gene expression is regulated by noncoding RNAs, including miRNAs. MiRNAs, which are endogenously expressed in mammals [32–34], are 18–22-nucleotide (nt), small noncoding RNAs that are evolutionarily conserved and negatively regulate the expression of target mRNAs mainly by repressing translation or, in some cases, by cleaving mRNA transcripts [35, 36]. miRNAs play a key role in many biological processes by regulating gene expression at the posttranscriptional level. This will result in a change in the expression levels of the set of target genes by approximately 15–40% at the mRNA and protein levels [36]. miRNAs act as sponges to adsorb circular RNAs and serve as bridges for ceRNAs. miRNAs can also compete with lncRNAs for binding. They play a key role in gene expression regulatory networks. Individual miRNAs exhibit unique spatiotemporal expression patterns in developing and cycling HFs [36]. Therefore, it is important to understand the role of miRNAs in HF development. Several miRNAs have been identified from livestock species. However, compared with the results reported livestock such as goats (267 precursors, 436 mature [CHIR_2.0]) and cows (1064 precursors, 1025 mature [Btau_5.0.1]), the number of miRNAs identified in sheep is quite low (106 precursors, 153 mature [Oar_v4.0]), particularly in skin tissues. Recently, several studies have identified genes and miRNAs involved in HF development in sheep [37–40] and goats [41–43].

miR-205 and miR-203 are squamous epithelial miRNAs [44–46]. During skin development, Yi et al. showed that miR-205 is highly enriched in epithelial progenitors and stem cells. In addition, they found that miR-205 regulates PI(3)-kinase signaling and acts as an HF stem cell activator with a crucial role in HF morphogenesis [44]. Moreover, they showed that miRNA-203 is induced during stratification and differentiation in the skin [45]. miR-125b acts as a repressor of the differentiation of HF stem cells and is therefore crucial to the onset of anagen [47, 48]. The miRNAs miR-31 [49] and miR-214 [50] are abundantly expressed in proliferating hair matrix keratinocytes and the outer root sheath during anagen. During the hair cycle, miR-31 modulates gene expression programs in the skin and HFs. miR-31 modulates hair growth at least partly by modulating BMP and FGF signaling pathways [49]. miR-214 inhibits hair growth by inhibiting the Wnt and Shh signaling pathways [50]. miR-24 is involved in the regulation of differentiation programs during HF development and is predominantly expressed in differentiated keratinocytes of the inner root sheath [51]. When miR-24 is overexpressed in proliferating cells during skin morphogenesis, abnormal HF development is associated with reduced proliferation and premature differentiation [52]. HF and skin miRNA databases were enriched by these studies, and our understanding of miRNA regulation in skin and HFs was improved. However, the mechanisms by which miRNAs regulate HF development in fine-wool sheep are not well understood.

In this study, to elucidate the molecular mechanisms of HF development in sheep, we sequenced miRNAs across six important HF development stages (i.e., E65, E85, E105, E135, D7, D30) in the skin tissue of 18 Merino sheep. To understand the functions of miRNAs and their target genes in HFs, we constructed an miRNA–mRNA regulatory network including the results of previous mRNA analyses conducted by our research group. Additionally, the targeting relationships between miRNAs and mRNAs were verified with a dual-luciferase reporter gene system, and RT–qPCR and Western blotting verified the overexpression/inhibition of miRNAs in sheep skin dermal fibroblasts. This work provides new insight into the miRNA and mRNA interaction networks involved in HF development and will potentially benefit wool quality control in the wool industry.

## Results

### miRNA sequencing and quality assessment results

All sequencing data for each small RNA library are presented in Additional file 1: Table S1. We mapped these clean reads using the Ensembl sheep genome to further elucidate the possible mechanisms underlying the diversity of small RNAs involved in HF development. Mapping the total reads to parts of the genome resulted in the mapping of more than 80% of the clean reads in the 18 libraries (Additional file 1: Table S1), and the length distribution of small RNAs differed among the eighteen libraries. We filtered raw data, and the results showed that the coverage of clean miRNA reads (Q30) was more than 75% (Additional file 1: Table S1). In all libraries, most of the clean reads were 22 nucleotides long (Additional file 2: Fig. S1b). The sequencing depth and coverage of the samples met the requirements of this experiment and indicated that they could be used for subsequent experiments.

### RT–qPCR validation of miRNA-seq

**Fig. 1:**
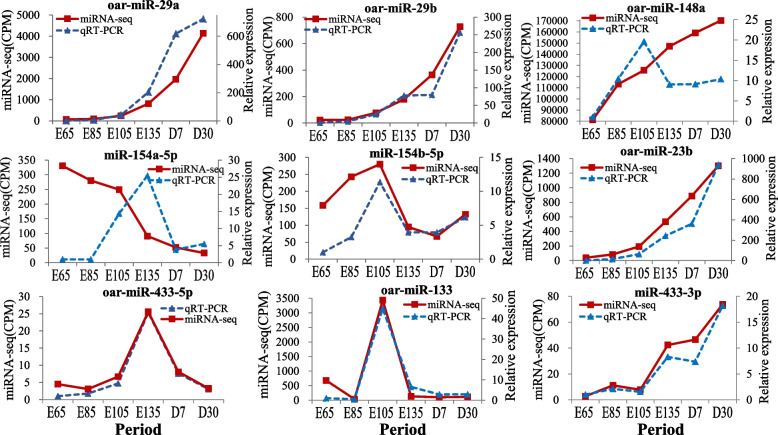
Results of RT–qPCR and miRNA-seq. The x-axis represents the time period, and the y-axis represents relative expression

We compared the RT–qPCR results with the miRNA-seq results (Fig. 1). We found by RT–qPCR that the skin expression levels of miR-148a in three periods (E135, D7 and D30) and miR-154a-5p in four periods (E65 to E135) were inconsistent with the sequencing results. The RT–qPCR expression levels of the remaining seven miRNAs were consistent with the miRNA-seq results. Therefore, we concluded that the sequencing results were accurate and reliable.

### Identification of differentially expressed miRNAs

**Fig. 2:**
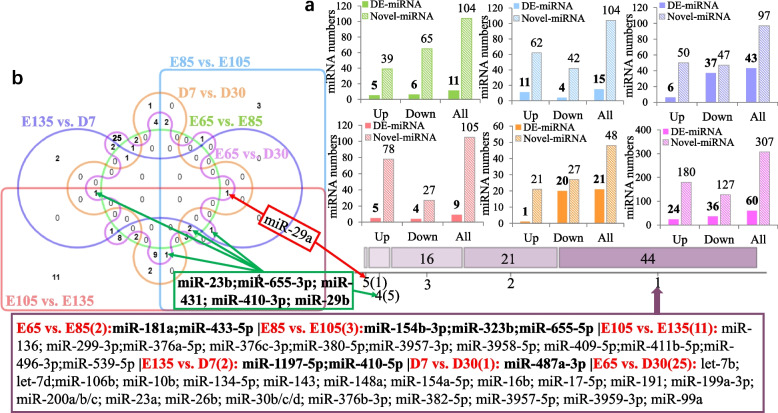
**a** The DE-miRNAs and novel DE-miRNAs between groups were obtained via pairwise comparisons; Up indicates a significantly upregulated miRNA; Down indicates a significantly downregulated miRNA. **b** Venn diagram of DE-miRNAs

A total of 87 DE-miRNAs and 446 novel DE-miRNAs were identified in the six HF development stages in SBM sheep. Previous research by our group identified 7879 DE-mRNAs [53]. A total of 11 DE-miRNAs and 104 novel DE-miRNAs were identified in E65 vs. E85, among which 5 DE-miRNAs were upregulated and 6 were downregulated, while 39 novel DE-miRNAs were upregulated and 65 were downregulated (Fig. 2a). When E85 was compared to E105, 15 DE-miRNAs (including 11 upregulated and 4 downregulated DE-miRNAs) and 104 novel DE-miRNAs (including 62 upregulated and 42 downregulated novel DE-miRNAs) were identified. In total, 43 DE-miRNAs (6 upregulated and 37 downregulated) and 97 novel DE-miRNAs (50 upregulated and 47 downregulated) were found between E105 and E135. Between E135 and D7, 9 DE-miRNAs (5 upregulated and 4 downregulated) and 105 novel DE-miRNAs (78 upregulated and 27 downregulated) were identified. When D7 was compared to D30, 21 DE-miRNAs (1 upregulated and 20 downregulated) and 48 novel DE-miRNAs (21 upregulated and 27 downregulated) were identified. The E65 vs. D30 comparison revealed the greatest number of DE-miRNAs (60), and E135 vs. D7 showed the fewest DE-miRNAs (only 9). These results indicated considerable differences between the beginning of HF development and postnatal development. Venn diagram analysis (Fig. 2b) indicated that miR-29a was differentially expressed in the five groups (except E65 vs. E85). miR-23b, miR-655-3p, miR-431, miR-410-3p and miR-29b were differentially expressed in four different periods. Likewise, 44 DE-miRNAs were specifically expressed in only one period, among which E65 vs. E85 showed 2 DE-miRNAs (miR-181a and miR-433-5p, respectively). There were 3 miRNAs (miR-154b-3p, miR-323b and miR-655-5p) specifically expressed in E85 vs. E105. Eleven miRNAs (miR-136, miR-380-5p, miR-411b-5p, etc.) were specifically expressed E105 vs. E135. Two miRNAs (miR-1197-5p and miR-410-5p) and one miRNA (miR-487a-3p) were specifically expressed in E135 vs. D7 and D7 vs. D30, respectively. E65vs. D30 showed the greatest number of specifically expressed miRNAs (25).

### Enrichment analysis of miRNA target genes

**Fig. 3:**
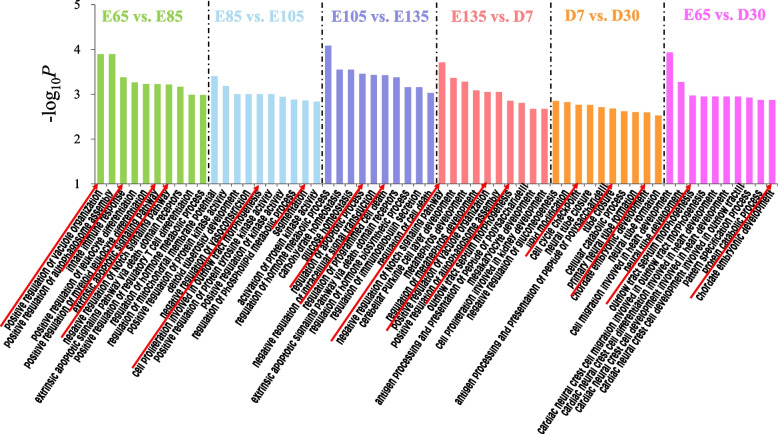
Top 10 GO classification statistics of miRNA target genes in different stages of hair follicle morphogenesis

To further elucidate the functions of the DE-miRNAs, we also examined the top 10 GO functional enrichment biological process (BP) (Fig. 3), cellular component (CC) and molecular function (MF) terms (Additional file 3: Fig. S2) and KEGG pathways (Additional file 4: Fig. S3) of target genes in adjacent comparison groups. In E65 vs. E85, the enriched GO terms were mainly related to the positive regulation of vacuole organization, innate immune response, extrinsic apoptotic signaling pathway and negative regulation of the Notch signaling pathway. The GO terms of E85 vs. E105 were enriched in cell proliferation involved in outflow tract morphogenesis and segmentation. In E105 vs. E135, the terms were mainly concentrated in the regulation of the apoptotic process and the regulation of programmed cell death. In E135 vs. D7, the terms were mainly enriched in BPs related to the negative regulation of the Notch signaling pathway, positive regulation of vacuole organization, and outflow tract septum morphogenesis. In D7 vs. D30, the terms were mainly enriched in cell cycle checkpoint, tube closure and chordate embryonic development. In the comparative analysis between 30 days after birth (D30) and the initial stage of HF development (E65), the target genes were enriched in outflow tract septum morphogenesis and chordate embryonic development. In summary, the target genes identified in E65 vs. E85 were directly related to cellular immunity and apoptosis. The target genes identified in E105 vs. E135 were significantly correlated with apoptosis. The target genes identified in E135 vs. D7 were related to kidney development, and those in E65 vs. D30 were related to cardiac and neurological development.

We performed KEGG enrichment analysis and found that the target genes were mainly enriched in the proteasome, the AMPK signaling pathway, protein processing in the endoplasmic reticulum and other pathways. Similarly, they were enriched in the neomycin, kanamycin and gentamicin biosynthesis pathways (Additional file 4: Fig. S3).

### DE-mRNA and DE-miRNA cluster analysis

**Fig. 4:**
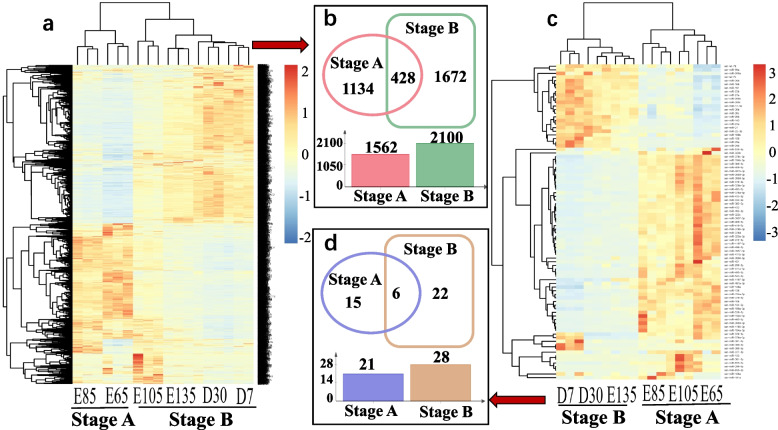
**a** Heatmap of DE-mRNAs. **b** Venn diagram of DE-mRNAs. **c** Heatmap of DE-miRNAs. **d** Venn diagram of DE-miRNAs

To screen mRNAs and miRNAs according to heatmap analysis, the DE-mRNAs identified at the first two time points (E65 and E85) and the last four time points (E105, E135, D7 and D30) were combined as Stage A and Stage B DE-mRNAs, respectively (Fig. 4a). Similarly, the DE-miRNAs from the first three time points (E65, E85 and E105) and the last three time points (E135, D7 and D30) were combined as Stage A and Stage B DE-miRNAs, respectively (Fig. 4c). Then, we generated a Venn diagrams for Stage A and Stage B. The numbers of Stage A DE-mRNAs and DE-miRNAs were 1562 and 21, respectively, and the numbers of Stage B DE-mRNAs and DE-miRNAs were 2100 and 28, respectively. The total numbers of DE-mRNAs and DE-miRNAs in the two groups were 428 and 6. The DE-mRNAs that appeared in both groups were eliminated, and the remaining 1134 DE-mRNAs were identified as candidate genes associated with the respective HF development stage (Stage A). The remaining 1672 DE-mRNAs are candidate genes associated with the HF maturation stage (Stage B) (Fig. 4b). The remaining 15 DE-miRNAs were candidates associated with stage A. The remaining 22 DE-miRNAs were candidates associated with stage B (Fig. 4d).

### Enrichment analysis of mRNA Stage A and Stage B

**Fig. 5:**
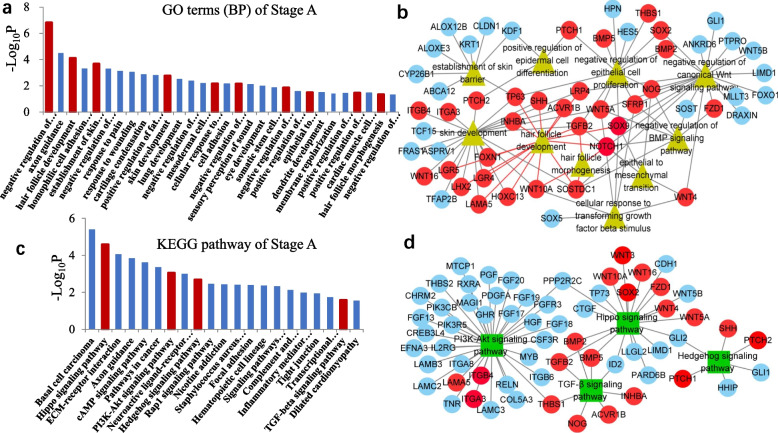
**a** Statistical map of GO classifications in Stage A. **b** Analysis of GO networks related to HF development (triangles represent GO terms, circles represent genes). **c** Statistical map of signaling pathways s in Stage A. **d** Analysis of KEGG networks related to hair follicle development (squares represent KEGG pathways, circles represent genes)

To further study the biological functions of DE-mRNAs related to the development of follicles in SBM during the fetal period, GO functional analysis and KEGG pathway analysis were performed on the stage A DE-mRNAs (Fig. 5). In the BP category, the DE-mRNAs were significantly enriched in the negative regulation of the canonical Wnt signaling pathway, HF development, establishment of the skin barrier, skin development, the cellular response to transforming growth factor beta stimulus, negative regulation of the BMP signaling pathway, negative regulation of epithelial cell proliferation, epithelial-to-mesenchymal transition, positive regulation of epidermal cell differentiation and HF morphogenesis (Fig. 5a). These BPs have been reported to be associated with HF morphogenesis, skin development or even hair formation. We visualized the genes involved in these BPs and found many DE-mRNAs. However, we focused on the 30 most meaningful genes (e.g., *TGFβ2, NOTCH1, WNT10A, ACVR1B, SOSOTCD1, WNT4,* and *BMP2*) (Fig. 5b). It is presumed that the initiation of HF development is mainly driven by the above factors.

A list of significant pathways was obtained via the KEGG analysis of the 1134 DE-mRNAs in Stage A (Fig. 5c). Four significant pathways were identified: the Hippo signaling pathway, the PI3K-Akt signaling pathway, the TGF-β signaling pathway and the Hedgehog signaling pathway. These pathways have also been reported to be significant in HF development in previous studies. We further analyzed the network of KEGG pathways related to HF development in Stage A and identified 20 genes, including *TGFβ2, WNT10A, PTCH1, BMP2, ACVR1B*, *WNT5A, WNT16, LAMA5, WNT10A,* and *WNT4*, that may be involved in HF development (Fig. 5d). In summary, we identified 19 genes (*LAMA5, ITGB4, ITGA3, THBS1, NOG, ACVR1B, INHBA, TGFβ2, BMP2, BMP5*, *WNT10A*, *SOX2*, *WNT16*, *WNT14*, *WNT5A*, *PTCH1*, *PTCH2*, *SHH*, and *FZD1*) that play a key role in Stage A of HF development.

**Fig. 6:**
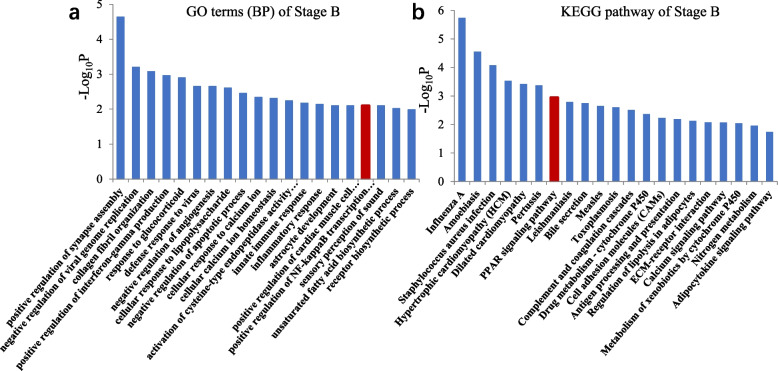
**a** Statistical map of GO classifications in Stage B. **b** Diagram of signaling pathways in Stage B

A list of significant pathways was obtained via the KEGG analysis of 1672 DEGs in Stage B (Fig. 6). We were surprised to find that only one biological process reported to be associated with HF development was enriched in Stage B: the positive regulation of NF-kappa B transcription factor activity (Fig. 6a). Among the identified pathways, the PPAR signaling pathway has been best studied in the context of HF development (Fig. 6b). Our study suggested that the genes in this pathway are associated with the late stage of HF development and maturation.

### Analysis of miRNA-mRNA and miRNA target DE-mRNA networks

**Fig. 7:**
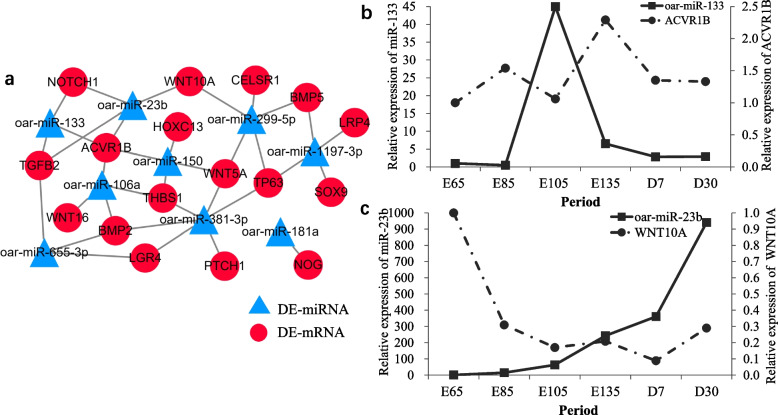
**a** DE-mRNA and DE-miRNA networks in Stage A. **b** Relative expression of miR-133 and ACVR1B in skin tissues at different stages of hair follicle morphogenesis. **c** Relative expression of miR-23b and WNT10A in skin tissues at different stages of hair follicle morphogenesis

Predicted target genes were combined with transcriptome profiling data to construct an mRNA–miRNA network associated with sheep HF growth and development. Significant mRNA–miRNA pairs in Stage A were selected to identify a total of 17 target genes of the 9 known sheep DE-miRNAs. The mRNA–miRNA network is shown in Fig. 7a.

To understand the roles of mRNAs and miRNAs in controlling skin morphogenesis and HF development, the expression of miR-133 and miR-23b and their target genes, ACVR1B and WNT10A, was evaluated in the skin of sheep at different stages of embryonic development and at postnatal D7 and D30. A low level of miR-133 expression was detected in embryonic skin during the onset of HF development from E65 to E85; its expression was significantly increased in E105 skin and then decreased significantly by E135. ACVR1B exhibited the opposite expression pattern (Fig. 7b). miR-23b expression gradually increased and was maximal on D30, whereas WNT10A expression showed the opposite pattern from E65 to E105 (Fig. 7c). Taken together, these findings suggest that miR-133 targets ACVR1B, while miR-23b targets WNT10A. To evaluate the targeting relationships between these miRNAs and genes, we constructed two luciferase reporter vectors, psiCHECK2-WNT10A and psiCHECK2-ACVR1B, for the transfection of 293 T cells.

### Validation of miR-133 and miR-23b functions in sheep dermal fibroblasts

**Fig. 8:**
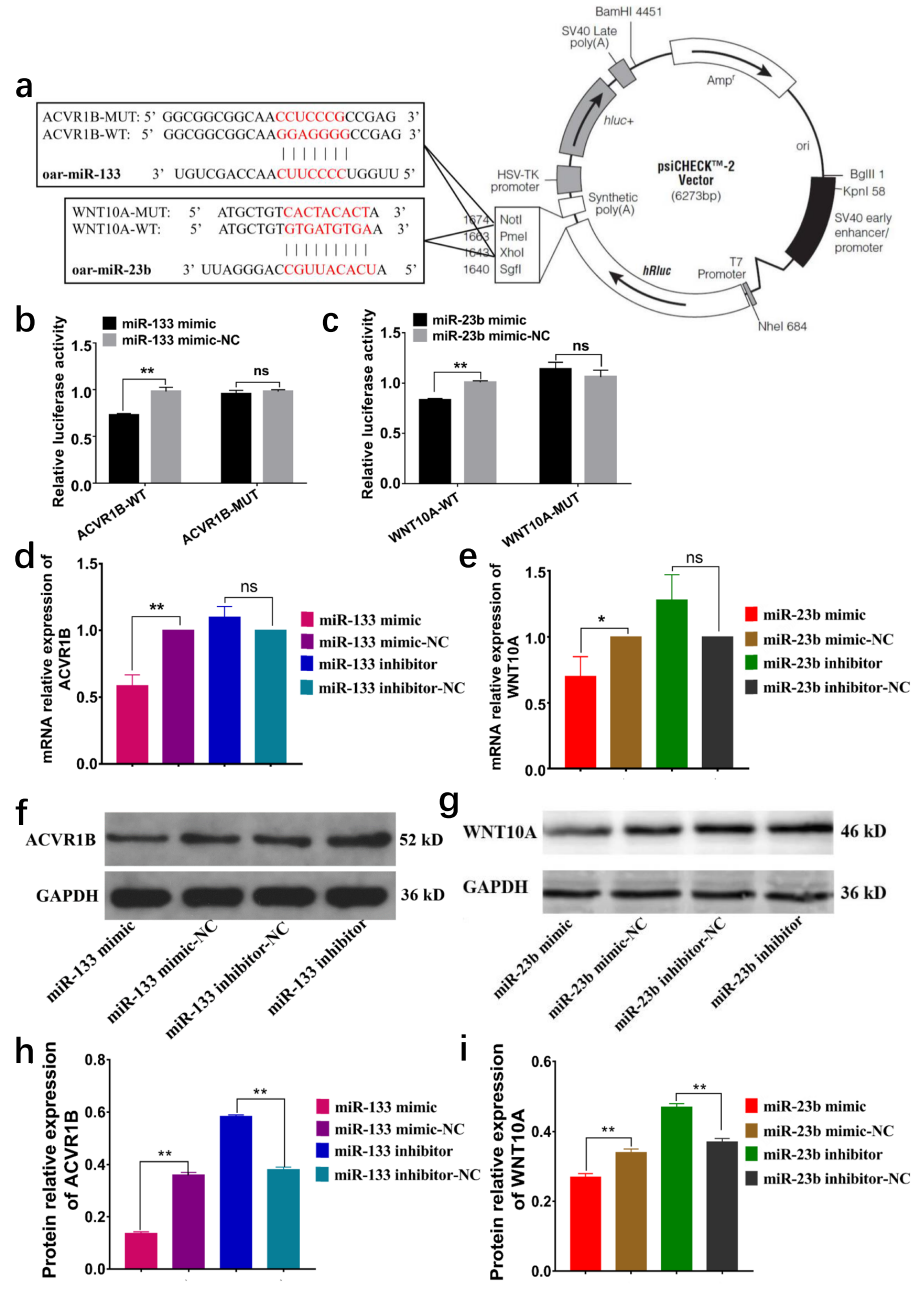
miR-133 could target and regulate ACVR1B, and miR-23b could target and regulate WNT10A. **a** The predicted sites of miR-133 binding to ACVR1B and miR-23b binding to WNT10A. **b** A luciferase reporter gene assay revealed that the overexpression of miR-133 significantly quenched wild-type ACVR1B fluorescence. **c** A luciferase reporter gene assay revealed that the overexpression of miR-23b significantly quenched wild-type WNT10A fluorescence. **d** RT–qPCR quantification indicated that the overexpression of miR-133 significantly reduced the mRNA expression level of ACVR1B. **e** RT–qPCR quantification indicated that the overexpression of miR-23b significantly reduced the mRNA expression level of WNT10A. **f** Western blot analysis indicated that the overexpression of miR-133 significantly reduced the protein expression level of ACVR1B. **g** Western blot analysis indicated that the overexpression of miR-23b significantly reduced the protein expression level of WNT10A. **h** Analysis of the relative protein expression of ACVR1B. **i** Analysis of the relative protein expression of WNT10A. **p < 0.01, *p < 0.05, ns *p* > 0.05

To validate ACVR1B and WNT10A as direct targets of miR-133 and miR-23b, respectively, ACVR1B and WNT10A luciferase reporters were constructed with wild-type and mutated ACVR1B and WNT10A 3’-untranslated regions (UTRs) s (Fig. 8a). These reporters were cotransfected with miR-133 and miR-23b mimics or a negative control (mimic-NC) into 293 T cells. Luciferase reporter activity was measured in dual luciferase assays. As shown in Fig. 8, luciferase activity significantly decreased when cells were cotransfected with miR-133 and miR-23b mimics compared to the transfection of either mutant or empty controls. The overexpression of miR-133 and miR-23b mimics decreased the luciferase activity of psiCHECK2-ACVR1B (Fig. 8b) and psiCHECK2-WNT10A (Fig. 8c).

We verified the network related to ACVR1B and WNT10A and showed that ACVR1B and WNT10A are target genes of miR-133 and miR-23b, respectively. To determine whether ACVR1B and WNT10A could be regulated by miR-133 and miR-23b, the mRNA levels of ACVR1B and WNT10A in sheep dermal fibroblasts transfected with miR-133 and miR-23b mimics and inhibitors were determined by RT–qPCR. The miR-133 and miR-23b mimics dramatically decreased the mRNA levels of ACVR1B and WNT10A, while miR-133 and miR-23b inhibitors increased ACVR1B (Fig. 8d) and WNT10A (Fig. 8e) mRNA levels in sheep dermal fibroblasts. These results were consistent with the observed decrease in WNT10A expression (Fig. 8c). To further test whether ACVR1B and WNT10A could be regulated by miR-133 and miR-23b, the protein levels of ACVR1B and WNT10A in sheep dermal fibroblasts transfected with miR-133 and miR-23b mimics and inhibitor were determined by Western blotting. The results showed that miR-133 and miR-23b mimics dramatically decreased the protein levels of ACVR1B and WNT10A, while miR-133 and miR-23b inhibitors increased ACVR1B (Fig. 8f, h, Additional file 5: Fig S4) and WNT10A (Fig. 8g, i, Additional file 5: Fig S4) protein levels in sheep dermal fibroblasts. Furthermore, the results were in agreement with the decreased expression of ACVR1B and WNT10A (Fig. 8d, e).

## Discussion

Microanatomy and cellular activity are dramatically altered during HF development and regeneration and are controlled by multiple signaling pathways, transcription factors and epigenetic regulators, including miRNAs. In the different HF cell lineages, miRNAs and their targets form remarkably diverse regulatory networks that play vital roles in gene expression programming. Andl and Botchkareva [36] summarized the roles of miRNAs in the control of HF development, cycling and hair pigmentation and presented future directions for this exciting and rapidly growing area of research. Through cell-specific or tissue-specific interference with miRNA biogenesis, it has been proven that miRNAs play indispensable roles in development and organogenesis. During embryonic development, the structural surface ectoderm-specific deletion of the miRNA processor Dicer or DgCr8 in the skin leads to serious abnormalities in HF development, characterized by the inability of HFs to invaginate into the dermis, leading to major structural defects and an inability to produce hair stems [3, 54]. Of course, miRNAs are essential not only for the development of HFs but also for postnatal HF growth. When Drosha or Dicer is deleted after HF initiation, fully developed HFs show a large number of apoptotic cells and structural changes in hair stems in the hair matrix, ultimately leading to the degradation of the HF [55]. These studies provide significant evidence that the global deletion of miRNAs in the embryonic epithelium significantly changes the molecular interaction between epithelial cells and dermal cells, resulting in the failure to form normal HFs, and show that miRNAs are necessary to control proliferation and apoptosis in adult HFs.

The skin sends signals to initiate hair bud formation, and the bud releases signaling factors that induce fibrotic cells to form dermal papillae that regulate keratinocyte activity [28]. Fibroblasts are major mesenchymal cells that deposit collagen and elastic fibers in the extracellular matrix (ECM); even within a single tissue, fibroblasts exhibit considerable functional diversity [29]. There are two distinct lines of fibroblasts that form the connective tissue of the skin. One forms the upper dermis, including the dermal papilla that regulates hair growth and the arrector pili muscle, which controls piloerection. The upper dermis is required for HF formation [28, 29].

The initiation of HFs involves a series of signaling pathways that link epidermal cells and the dermal papillae, such as the Wnt/β-catenin [56, 57], TGF-β [58] and Hippo signaling pathways. WNT, Hippo and TGF-β signals are required for the initiation of HF development. The current study also revealed that the above signaling molecules are expressed in sheep skin during the onset of secondary HF development. Based on the results of our study, we consider the DE-mRNAs and miRNAs of Stage A to have more practical significance in HF development than those of Stage B. E65, E85 and E105 represent the key periods for the formation and differentiation of PFs, SFs and SDs. We performed a combined analysis of the mRNA and miRNA sequencing results and ultimately identified 9 DE-miRNAs and 17 DE-mRNAs with targeting relationships in Stage A. Combined with existing research reports, our DE-mRNA enrichment analysis results revealed that the ACVR1B gene plays a key role in the TGF-β signaling pathway. WNT10A is expressed in the Wnt and Hippo signaling pathways. Therefore, we believe that ACVR1B and WNT10A play key roles in the initiation of HF development. Finally, we selected ACVR1B and WNT10A and their targeted miRNAs for subsequent validation. However, the dual luciferase reporter gene results showed that miR-23b and ACVR1B did not present a targeted regulatory relationship. The in vivo functions of ACVR1B have been difficult to study because ACVR1B knockout mice die during embryogenesis. Mice with conditional disruption of ACVR1B display various degrees of hairlessness at postnatal day 5, and the phenotype becomes more severe with age [29]. HFs that develop during morphogenesis are later disrupted by delays in hair cycle re-entry and the failure of cycling and regrowth of the hair shaft and the inner root sheath, resulting in severe hair loss [29]. This study demonstrates a specialized role of ACVR1B in hair cycling as well as HF development. Therefore, ACVR1B signaling is required for both HF development and cycling. In our study, we found that miR-133 could target and regulate ACVR1B. In dermal fibroblasts, the overexpression of miR-133 significantly reduced the mRNA and protein expression levels of ACVR1B.

Andl [59] et al. used a secreted inhibitor of essential WNT coreceptors and showed that signaling by canonical WNT proteins is required for the initiation of all types of HFs. These results demonstrate that the actions of canonical WNT proteins in the skin precede the localized expression of genes that regulate HF placode formation and indicate that canonical WNT signaling is required for the generation of, or the initial response to, the first dermal message. Wu et al. [60] showed that WNT10B can act via the Wnt/β-catenin signaling pathway to inhibit HF development in vitro. Although these Wnts are expressed in the epidermal component of HFs and are candidate mediators of this activity in vivo, it is possible that another Wnt acting through the same signal transduction pathways may normally serve this function during the hair cycle. Reddy et al. [61] found that FZD1 expression was correlated with WNT10A and WNT10B expression in the placode. They suggested that canonical WNT signaling in the placodes and the dermal condensate of developing HFs is likely activated by WNT10A and WNT10B expressed in and secreted from epithelial cells that then bind to FZD1 expressed in the placode epithelium and dermal condensate. WNT10A and WNT10B are continuously present in the follicular epithelium at later germ and bulbous peg stages. Similarly, they are strongly expressed in the epithelial cone surrounding the dermal papilla during the bulbous peg stage. However, Wnt signaling through the β-catenin pathway is sufficient to maintain, but not restore, the anagen-phase characteristics of dermal papilla cells [62]. It was shown that WNT10A is expressed in the outer root sheath, inner root sheath, matrix and hair shaft of anagen follicles in rats [57]. The characterization of the molecular pathways controlling differentiation and proliferation in mammalian HFs is central to our understanding of the regulation of normal hair growth, the foundations of hereditary hair loss diseases and the origins of follicle-based tumors. Millar et al. [63] demonstrated that the expression of WNT10A, WNT11, and β-catenin was increased at days 14 and 21 post wounding, when tissue remodeling occurred. In the epithelial tongue and neoepidermis, the WNT10A signal contributes to cutaneous wound repair by stimulating ECM maturation during the initial stage of healing [64]. WNT10A expression, especially in fibroblasts, can be crucial for achieving various potentially beneficial effects in wound healing [65]. Studies have also shown that WNT10A plays an important role in the pathogenesis of idiopathic pulmonary fibrosis via TGF-β activation and may be a sensitive predictor of the onset of an acute exacerbation of idiopathic pulmonary fibrosis [66]. In our study, we found that miR-23b could target and regulate WNT10A. In dermal fibroblasts, the overexpression of miR-23b significantly reduced the mRNA and protein expression levels of WNT10A.

## Conclusion

In summary, we performed miRNA-seq of the miRNAs in six developmental stages of SBM HFs and conducted a bioinformatic analysis. We identified a total of 87 DE-miRNAs and 446 novel DE-miRNAs. We further performed mRNA–miRNA coanalysis in the six developmental stages of SBM HFs. We found that sheep HF development could be divided into two developmental stages (Stage A and Stage B). The mRNAs (E65 and E85) and miRNAs (E65 to E105) of stage A were closely related to HF morphogenesis. We found miRNAs and genes associated with hair follicular development, providing a theoretical basis for molecular breeding for the production of fine-wool sheep. Many more studies are needed to further delineate the significance of functional miRNA–mRNA interactions in HF biology. This research provides new insights into how the skin regulates cellular activity and cell fate decisions.

## Methods

### Animal selection and skin tissue preparation

SBM is a subtype of the Merino breed of sheep reared in China and is famous for the excellent quality and yield of its wool and its high survival rate. The tested individuals were selected from the sheep herd located at the Kechuang Animal Husbandry Breeding Center, Xinjiang, China. Twenty healthy SBM ewes were artificially inseminated with fresh sperm from the same SBM ram and then managed in the same flock. The day of insemination was designated embryonic day 0 (E0). Embryonic (E65, E85, E105 and E135) skin tissue collection has been described previously [53]. Skin tissue collection on postnatal days 7 and 30 (D7 and D30) has also been described previously [53]. Three biological replicates were generated for each of six developmental stages representing groups. All eighteen skin tissue samples were stored at − 80 °C.

### Total RNA isolation and sequencing

Total RNA was isolated from the tissues using TRIzol reagent (Invitrogen, Carlsbad, CA, USA). For miRNA sequencing, the integrity of the total RNA samples was assessed using an Agilent 2100 Bioanalyzer (Agilent Technologies Inc., USA), and samples with RNA integrity numbers (RINs) above 7.0 were used for sequencing. Paired-end libraries were synthesized by using the QIAseq miRNA Library Kit (Qiagen, Germany) following the QIAseq miRNA Library Kit Guide. The products were then purified and enriched via PCR to create the final cDNA library. Purified libraries were quantified with a Qubit® 2.0 fluorometer (Life Technologies, USA). Clusters were generated by using cBot with the library diluted to 10 pM and were then sequenced on the Illumina HiSeq Xten platform (Illumina, USA).

### Analysis of sequencing data

The sequence analysis of DE-mRNAs is detailed in another article we previously published [53]. miRNA-seq reads were filtered, and clean reads were mapped to the sheep (Ovis aries V3.1) mature miRNA database in miRBase (v21) and the Rfam database (ftp://selab.janelia.org/pub/Rfam) to match them with known long noncoding RNA, miRNA, ribosomal RNA, small nuclear RNA, small nucleolar RNA, and transfer RNA sequences. miRNA abundance was expressed as counts of exon model per million mapped reads (CPM). DE-miRNAs with |log2 (FC)| value > 1 and *p* value < 0.05 were considered significantly modulated. Clean reads that were not mapped to sheep mature miRNAs were then mapped to the Oar 3.1 version of the sheep genome sequence using Bowtie software, and the distribution of mapped reads on the chromosomes was calculated. Novel miRNAs were also predicted by using miRDeep2. In the mixed sequencing data of all samples of the studied species, various non-miRNA sequences were filtered out, and the remaining sequencing data (defined as clean miRNA reads) were used for the identification and structural prediction of new miRNAs. Predicting new miRNAs requires reference genome sequences. In this program, miRDeep software is used in combination with homologous miRNA sequences of closely related species, including the application of RNA secondary structure prediction software such as RNAfold, to predict and identify the mature forms (Star miRNA and mature miRNA) and precursors of new miRNAs. For each newly predicted miRNA, miRDeep software applies various evaluation methods to evaluate the prediction accuracy. The expression patterns of DE-miRNAs were measured by systematic cluster analysis to explore the similarities and compare the relationships between the different libraries.

### RT–qPCR validation of miRNA-seq results

To confirm the differential expression of genes revealed by miRNA-seq, a total of nine miRNAs identified as differentially expressed among different developmental stages were randomly chosen for RT–qPCR validation. Total RNA was extracted using TRIzol reagent (Invitrogen). A Poly(A) Tailing Kit (Ambion, Waltham, MA, USA) was used to add poly(A) tails to miRNAs. We used the PrimeScript™ RT Reagent Kit with gDNA Eraser (Takara, Dalian, China) and gene-specific primers or random primers to generate cDNA. RT–qPCR was performed in a CFX96™ Real-Time System (Bio-Rad, USA) using the miRcute Plus miRNA qPCR Kit (SYBR Green) (Tiangen, China). U6 snRNA was employed as an endogenous control for miRNA. In the miRNA RT‒qPCR assay, the thermal cycling conditions were 95 °C for 15 min, followed by 45 cycles at 94 °C for 20 s and 60 °C for 34 s. Three biological and technical replicates were conducted. The 2^−ΔΔCt^ method was used to determine relative miRNA abundance. Primer sequences are displayed in Additional file 6: Table S2.

### GO enrichment and KEGG pathway analysis

The GO (http://geneontology.org) and KEGG (http://www.genome.ad.jp/kegg/) databases of DAVID were used to perform functional annotations. DE-mRNAs identified in Stages A and B of HF development were analyzed. Based on the false discovery rate (FDR), we determined the GO terms and metabolic pathways significantly associated with the gene lists. An FDR ≤ 0.05 was applied to significant candidate genes associated with BPs or metabolic pathways.

### miRNA target gene prediction and construction of an interaction network

In animals, miRNA binds to the 3’- UTR of the target gene mRNA in an incomplete complementary pairing manner. The genes in Stage A related to HF morphogenesis obtained from the above enrichment analysis were predicted using the miRanda, TargetScan (http://www.targetscan.org), PicTar (https://pictar.mdc-berlin.de), RNAhybrid (https://bibiserv.cebitec.uni-biele), miRWalk (http://mirwalk.umm.uni-heidelberg.de/), RNA22 (http://cbcsrv.watson.ibm.com/rna22.html) and starBase (http://starbase.sysu.edu.cn/) tools to predict miRNA target genes. Then, more than three cross-target genes were selected as target mRNAs in the Venn analysis. mRNAs whose miRNA-targeted mRNAs overlapped with DE-mRNAs in phase A were selected as DE-mRNAs. Finally, Cytoscape was used for the visualization and analysis of the DE-mRNA and DE-miRNA networks.

### Cell culture and transfection

HEK-293 T cells were used for the dual-luciferase reporter gene assay and cultured at 37 °C in Dulbecco’s modified Eagle’s medium (DMEM) (Invitrogen, Carlsbad, CA, USA) supplemented with 10% fetal bovine serum (FBS) (Invitrogen, Carlsbad, CA, USA), 1.5 mM l-glutamine (Invitrogen, Carlsbad, CA, USA), 100 U/mL penicillin (Invitrogen, Carlsbad, CA, USA), and 100 mg/mL streptomycin (Invitrogen, Carlsbad, CA, USA) in a humidified incubator in an atmosphere containing 5% CO_2_ (Thermo, Waltham, MA, USA). We seeded the cells into 24-well plates, and when the cells reached 80% confluence, we used Lipofectamine™ 3000 (Invitrogen, CA) to cotransfect 293 T cells with the constructed double luciferase reporter gene vector (psiCHECK2-WNT10A-WT, psiCHECK2-WNT10A-MUT, psiCHECK2-ACVR1B-WT and psiCHECK2-ACVR1B-MUT) along with miR-23b and miR-133 (mimic and mimic-NC).

Sheep dermal fibroblasts were used to verify the effects of miR-23b and miR-133 on their targeted mRNAs. The skin tissue of healthy postnatal lambs at 7 days of age was collected at the Xinjiang Kechuang Breeding Center. Sheep skin was treated and cultured at 37 °C with 5% CO_2,_ and the medium was then changed every three days to observe cell growth in the culture dish to obtain primary fibroblasts. Cells were passaged when the cell confluency was more than 90%. When the cells reached to the fourth generation, they were inoculated into a 6-well plate and grown until the confluence rate reached 80%. We used Lipofectamine™ 3000 (Invitrogen, CA)-transfected fibroblasts with miRNA overexpression (miR-23b mimic and miR-133 mimic) or knockout (miR-23b inhibitor and miR-133 inhibitor) or a negative control (mimic-NC and inhibitor-NC) according to the manufacturer's instructions.

### Dual luciferase reporter gene assay

Luciferase reporters used to verify the targeting relationship between miRNAs and mRNAs were generated based on the psiCHECK2 vector (Promega). To construct psiCHECK2-WNT10A and psiCHECK2-ACVR1B, the complete 3’-UTRs of sheep WNT10A mRNA (676 nt, GenBank accession no. XM_004004935) and ACVR1B mRNA (2966 nt, GenBank accession no. XM_027967297), containing the putative miR-133 and miR-23b binding sites, were amplified and cloned into the psiCHECK2 vector. The luciferase reporter was cotransfected with miR-133 mimics, miR-23b mimics, and mimics-NC into 293 T cells by using Lipofectamine™ 3000 according to the manufacturer’s guidelines. Relative luciferase activity was measured with a Dual-Luciferase Reporter Assay System (Promega) and an Infinate M200 PRO microplate reader (Tecan, Shanghai, China).

### RT–qPCR and Western blotting of fibroblasts

RNA was extracted using an RNAsimple Total RNA Kit (Tiangen, China) and reverse transcribed into cDNA using a FastQuant RT Kit (Tiangen, China) after sheep dermal fibroblasts had been transfected for 36 h. RT–qPCR was used to quantify WNT10A and ACVR1B mRNA expression in the different treatment groups using TB Green Premix Ex Taq II (TaKaRa, Dalian, China) following the manufacturer's instructions. The RT‒qPCR experiments were conducted in triplicate, and the relative abundance of mRNA was determined using the 2^−ΔΔCt^ method. ANOVA was used for statistical comparison of more than two groups. All statistical analyses were conducted using SPSS 20.0 software (IBM Corp, Armonk, NY, USA). *P* < 0.05 was considered statistically significant.

For Western blot analysis, dermal fibroblast cells were lysed using RIPA Lysis Buffer II (Sangon, Shanghai, China). Protein concentrations in different groups were measured with a Pierce™ Rapid Gold BCA Protein Assay Kit (Thermo Scientific, Waltham, MA, USA). The proteins were separated by SDS–PAGE, transferred to polyvinylidene difluoride membranes, and probed with 1:1000 rabbit anti-WNT10A (ABclonal, A19090, China), 1:1000 rabbit anti-ACVR1B (ABclonal, A2279, China) and 1:1000 rabbit anti-GAPDH (ABclonal, A19056, China) antibodies and then with 1:1000 HRP goat anti-rabbit IgG (H + L) (ABclonal, AS014, China)-conjugated antibodies. All steps were carried out in accordance with the provided instructions. Signals were detected with an enhanced chemiluminescence (ECL) substrate kit (UltraSensitive) (Biosharp, Beijing, China). Proteins were detected and analyzed with the Wes Automated Western Blot Analysis System (Protein Simple, CA, US).

## Supplementary Information

**Additional file 1: Table S1.** Result Statistics of miRNA Genome Comparison.

**Additional file 2: Figure S1.** The distribution of the read from the sequencing data. (a) Read redundancy statistics of different samples. (b) Read length distribution of different samples.

**Additional file 3: Figure S2.** Analysis of miRNA target genes go enrichment at different stages of hair follicle development. (a) GO enrichment analysis of E65 vs. E85 target genes. (b) GO enrichment analysis of E85 vs. E105 target genes. (c) GO enrichment analysis of E105 vs. E135 target genes. (d) GO enrichment analysis of E135 vs. D7 target genes. (e) GO enrichment analysis of D7 vs. D30 target genes. (f) GO enrichment analysis of E65 vs. D30 target genes. where red is BP (biological processes), green is CC (Cellular components) and blue is MF (Molecular functions).

**Additional file 4: Figure S3.** Enrichment analysis of miRNA target gene KEGG pathway at different stages of hair follicle development.

**Additional file 5: Figure S4.** (a) Western blot level of ACVR1B (target strip in red box dimension). (b) Western blot level of GAPDH (target strip in red box dimension). (c) Western blot level of WNT10A (target strip in red box dimension). (d) Western blot level of ACVR1B (target strip in red box dimension). All the images of western blots are cut prior to hybridization with antibodies. Because the markers are marked on both sides of the glue, the notch in the upper right corner (upper left corner) is to mark the front and back.

**Additional file 6: Table S2.** Primer sequence.

## Data Availability

All miRNA-seq data generated in this study were submitted to the NCBI SRA database under BioProject No. PRJNA705552 (https://www.ncbi.nlm.nih.gov/bioproject/?term=PRJNA705552). All RNA-seq data generated in this study were submitted to the NCBI SRA database under BioProject No. PRJNA705554 ( https://www.ncbi.nlm.nih.gov/bioproject/?term=PRJNA705554).

## Abbreviations

HF: Hair follicle
miRNA: MicroRNA
mRNA: Messenger RNA
DE-miRNA: Differentially expressed miRNA
DE-mRNA: Differentially expressed mRNA
SBM: Subo merino
PF: Primary follicle
SF: Secondary follicle
SD: Secondary-derived follicle
TGF-β: Transforming growth factor beta
BMP: Bone morphogenetic protein
RT–qPCR: Reverse transcription quantitative real-time PCR
GO: Gene Ontology
KEGG: Kyoto Encyclopedia of Genes and Genomes
BP: Biological processes
ECM: Extracellular matrix
CPM: Counts of exon model per million mapped reads
FC: Fold-change
DAVID: Database for Annotation, Visualization, and Integrated Discovery
FDR: False discovery rate
3’ UTR: 3’-Untranslated region
