# Supplementary material for: Integrated analysis of miRNAs and mRNA profiling reveals the potential roles of miRNAs in sheep hair follicle development
Source: BMC Genomics. 2022 Oct 22;23:722. doi: 10.1186/s12864-022-08954-2 (PMC9588206; doi:10.1186/s12864-022-08954-2)
Supplement: Supplementary file 2 — Additional file 2: Figure S1. The distribution of the read from the sequencing data. (a) Read redundancy statistics of different samples. (b) Read length distribution of different samples. [file 12864_2022_8954_MOESM2_ESM.docx]

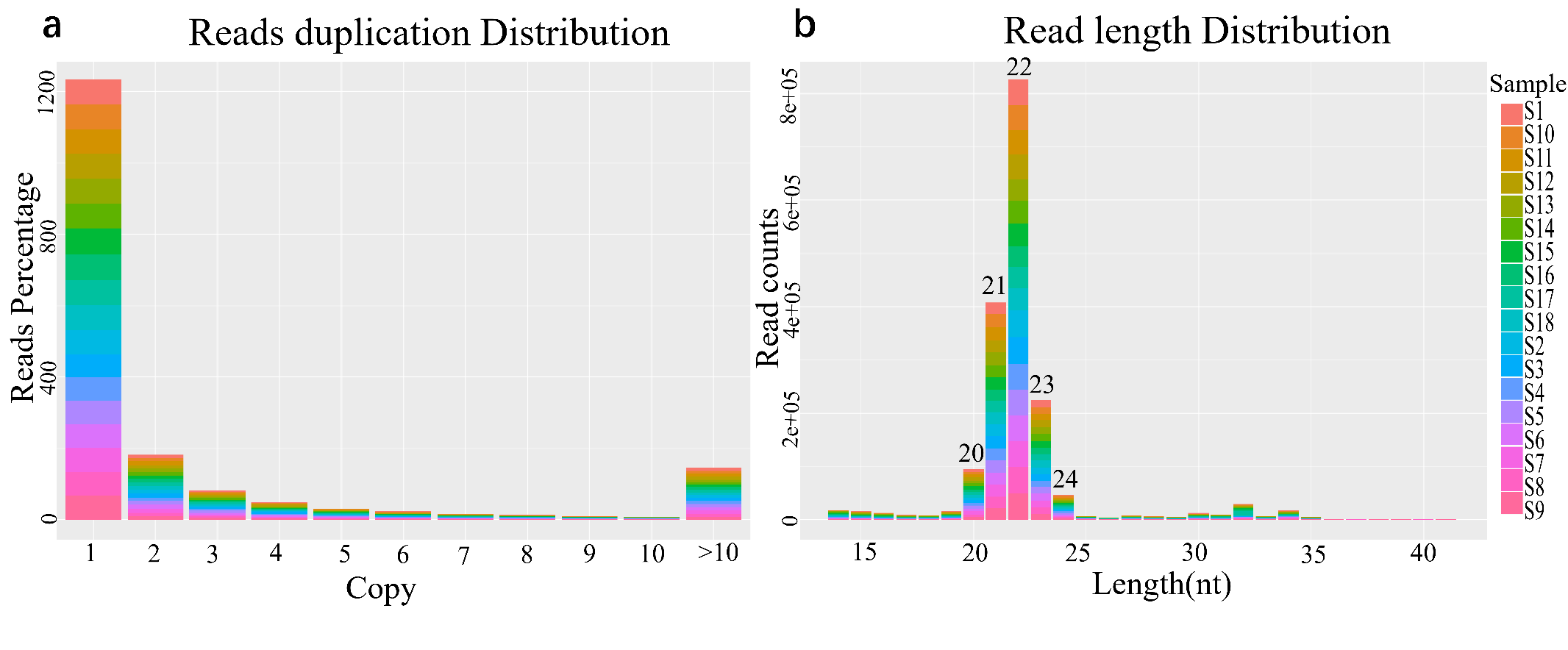


Figure S1. The distribution of the read from the sequencing data. (a) Read redundancy statistics of different samples. (b) Read length distribution of different samples.
