# Supplementary material for: Integrated analysis of miRNAs and mRNA profiling reveals the potential roles of miRNAs in sheep hair follicle development
Source: BMC Genomics. 2022 Oct 22;23:722. doi: 10.1186/s12864-022-08954-2 (PMC9588206; doi:10.1186/s12864-022-08954-2)
Supplement: Supplementary file 3 — Additional file 3: Figure S2. Analysis of miRNA target genes go enrichment at different stages of hair follicle development. (a) GO enrichment analysis of E65 vs. E85 target genes. (b) GO enrichment analysis of E85 vs. E105 target genes. (c) GO enrichment analysis of E105 vs. E135 target genes. (d) GO enrichment analysis of E135 vs. D7 target genes. (e) GO enrichment analysis of D7 vs. D30 target genes. (f) GO enrichment analysis of E65 vs. D30 target genes. where red is BP (biological processes), green is CC (Cellular components) and blue is MF (Molecular functions). [file 12864_2022_8954_MOESM3_ESM.pdf]

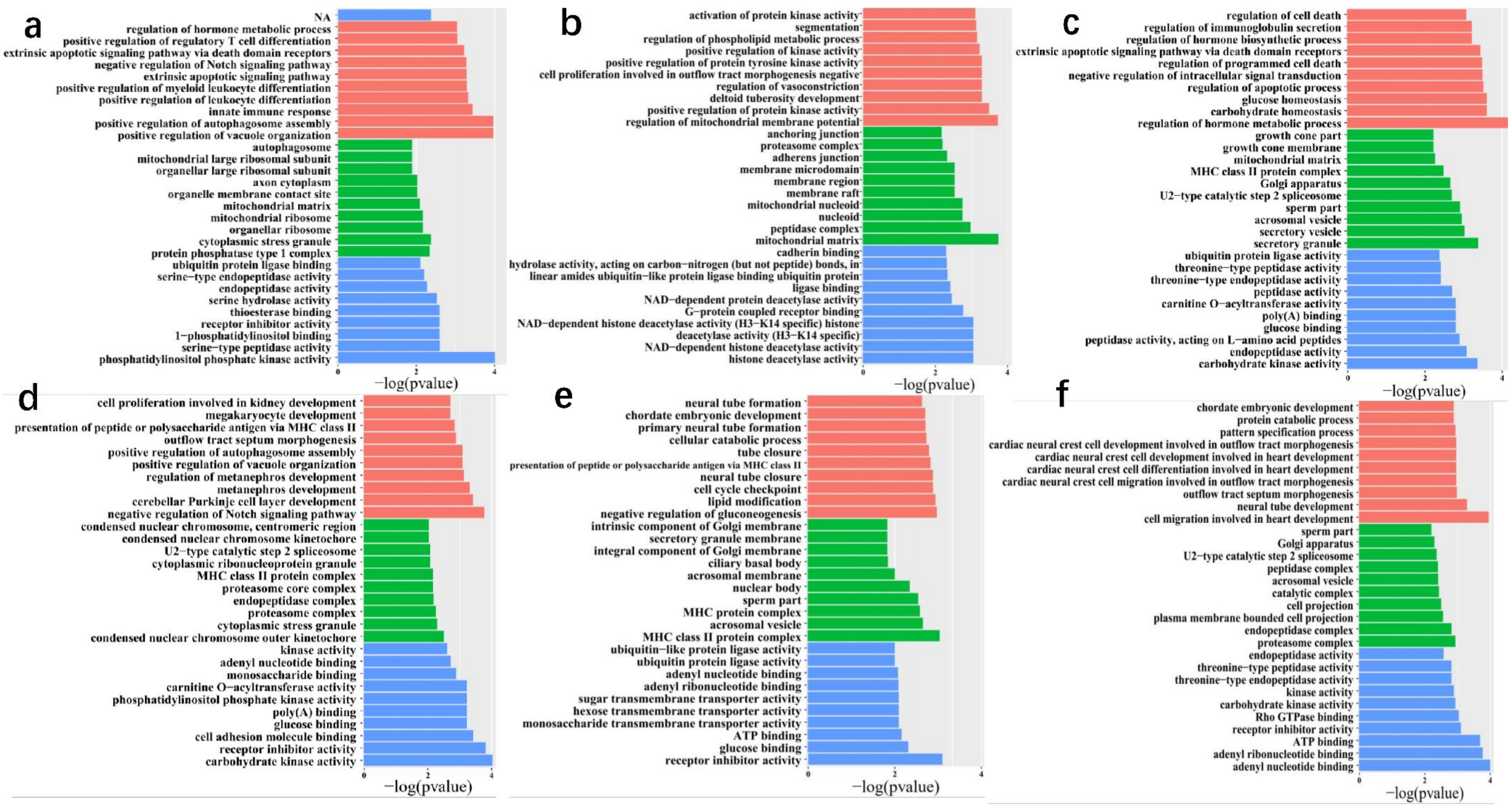

Figure S2. Analysis of miRNA target genes go enrichment at different stages of hair follicle development. (a) GO enrichment analysis of E65 vs. E85 target genes. (b) GO enrichment analysis of E85 vs. E105 target genes. (c) GO enrichment analysis of E105 vs. E135 target genes. (d) GO enrichment analysis of E135 vs. D7 target genes. (e) GO enrichment analysis of D7 vs. D30 target genes. (f) GO enrichment analysis of E65 vs. D30 target genes. where red is BP (biological processes), green is CC (Cellular components) and blue is MF (Molecular functions).
