# Supplementary material for: Integrated analysis of miRNAs and mRNA profiling reveals the potential roles of miRNAs in sheep hair follicle development
Source: BMC Genomics. 2022 Oct 22;23:722. doi: 10.1186/s12864-022-08954-2 (PMC9588206; doi:10.1186/s12864-022-08954-2)
Supplement: Supplementary file 4 — Additional file 4: Figure S3. Enrichment analysis of miRNA target gene KEGG pathway at different stages of hair follicle development. [file 12864_2022_8954_MOESM4_ESM.pdf]

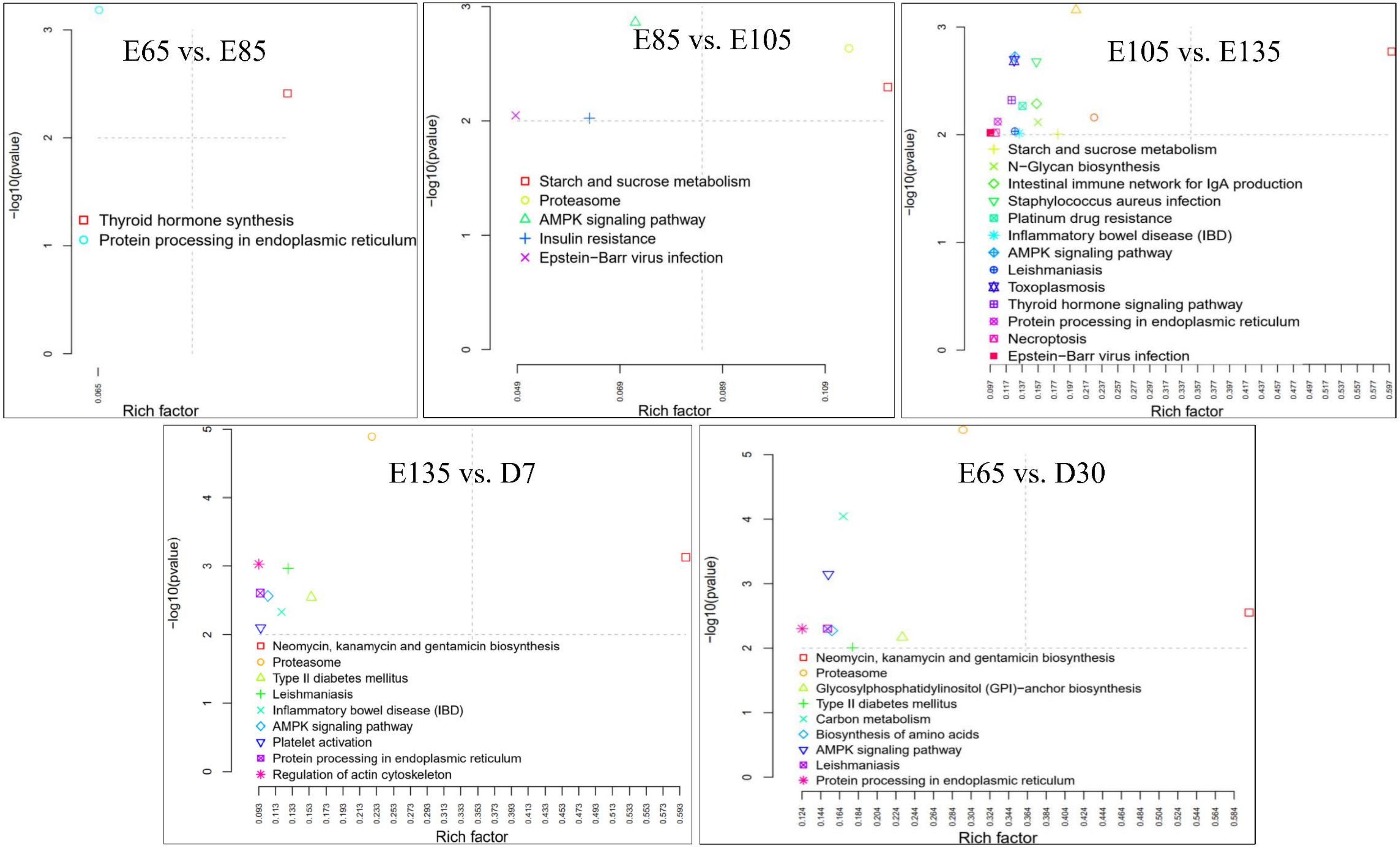

Figure S3. Enrichment analysis of miRNA target gene KEGG pathway at different stages of hair follicle development.
