# Supplementary material for: Integrated analysis of miRNAs and mRNA profiling reveals the potential roles of miRNAs in sheep hair follicle development
Source: BMC Genomics. 2022 Oct 22;23:722. doi: 10.1186/s12864-022-08954-2 (PMC9588206; doi:10.1186/s12864-022-08954-2)
Supplement: Supplementary file 5 — Additional file 5: Figure S4. (a) Western blot level of ACVR1B (target strip in red box dimension). (b) Western blot level of GAPDH (target strip in red box dimension). (c) Western blot level of WNT10A (target strip in red box dimension). (d) Western blot level of ACVR1B (target strip in red box dimension). All the images of western blots are cut prior to hybridization with antibodies. Because the markers are marked on both sides of the glue, the notch in the upper right corner (upper left corner) is to mark the front and back. [file 12864_2022_8954_MOESM5_ESM.pdf]

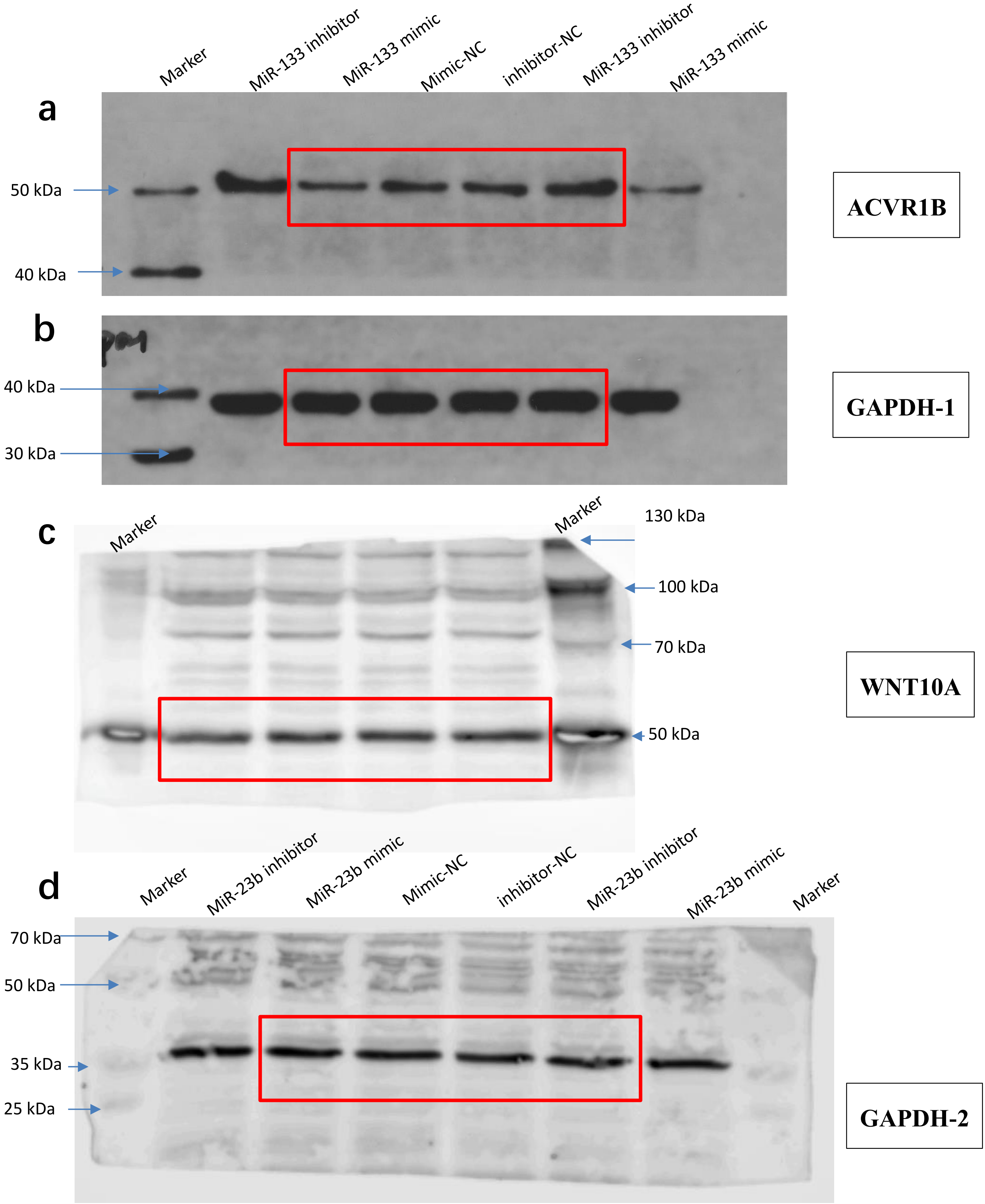

Figure S4. **(a)** Western blot level of ACVR1B (target strip in red box dimension). **(b)** Western blot level of GAPDH (target strip in red box dimension). **(c)** Western blot level of WNT10A (target strip in red box dimension). **(d)** Western blot level of ACVR1B (target strip in red box dimension). All the images of western blots are cut prior to hybridization with antibodies. Because the markers are marked on both sides of the glue, the notch in the upper right corner (upper left corner) is to mark the front and back.
